# Diffuse large B-cell lymphoma of the colon with synchronous liver metastasis: a rare case report mimicking metastatic colorectal adenocarcinoma

**DOI:** 10.1186/1471-2482-14-75

**Published:** 2014-10-07

**Authors:** Domenico Risio, Rossana Percario, Margherita Legnini, Francesco Caldaralo, Domenico Angelucci, Camilla Marinelli, Alberto D'Aulerio, Roberto Cotellese, Luca Napolitano, Paolo Innocenti

**Affiliations:** 1 Unit of General and Laparoscopic Surgery, “G. d’Annunzio” University, Via dei Vestini 31, 66100 Chieti, Italy; 2 Unit of Pathology, “G. d’Annunzio” University, Chieti, Italy

**Keywords:** Diffuse large B-cell lymphoma (DLBCL), Liver metastasis, Mantle cell lymphoma (MCL), Multiple lymphomatous polyposis (MLP), Primary colonic lymphoma (PCLs)

## Abstract

**Background:**

Primary colorectal lymphoma represents a rare minority among the colonic neoplasms. Early diagnosis is often difficult because of unspecific symptoms, with subsequent delays in diagnosis and management. We describe a rare case of colonic lymphoma presenting with synchronous liver metastasis.

**Case presentation:**

A 70-year-old male with a 6-mo history of vague abdominal pain, constipation and melena was referred to our hospital. Computed tomography scan of abdomen revealed the presence of a mass along the proximal ascending colon. Colonoscopy biopsy showed external compression of the cecum with two ulcerations of mucosa, but it was not consistent for a definitive diagnosis. Because the difficulties in the preoperative pathological diagnosis, the high risk of bowel obstruction and the correlated hemorrhagic risk, the patient underwent a right hemicolectomy associated with locoregional lymphadenectomy and liver resection.

The surgically resected right colon and liver tumors were all immunohistochemically diagnosed as diffuse large B-cell lymphomas (DLBCL). The patient refused any other antineoplastic treatment; he is alive and free of disease at 3 years after initial diagnosis.

**Conclusions:**

Primary colonic lymphomas represent a rare minority among the colonic neoplasms. Their correct pre-operative identification is crucial for the design of treatment. This case highlights the difficulty in diagnosing of primary colonic lymphoma. To our knowledge, this is the first report of a colonic lymphoma presenting with a colonic mass and a synchronous liver metastasis.

## Background

Primary colonic lymphoma (PCLs) is rare comprising 10–20% of all gastrointestinal lymphomas and less than 1% of large bowel malignancies. It is the third most common large bowel malignancy after adenocarcinoma and carcinoid [1]. Patients often present with vague and non-specific symptoms that subsequently lead to delay in diagnosis which often occurs after laparotomy and surgical resection. It is often associated with inflammatory bowel disease and immunosupression. Males are predominantly affected with highest incidence at the age of 50–70 years. Most PCLs have a B-cell lineage and are classified as diffuse large B-cell lymphomas (DLBCL) [2]. The optimal treatment for PCL is controversial. Here, we present an unusual case of diffuse large B-cell lymphomas of the cecum with synchronous liver metastasis.

## Case presentation

**Figure 1:**
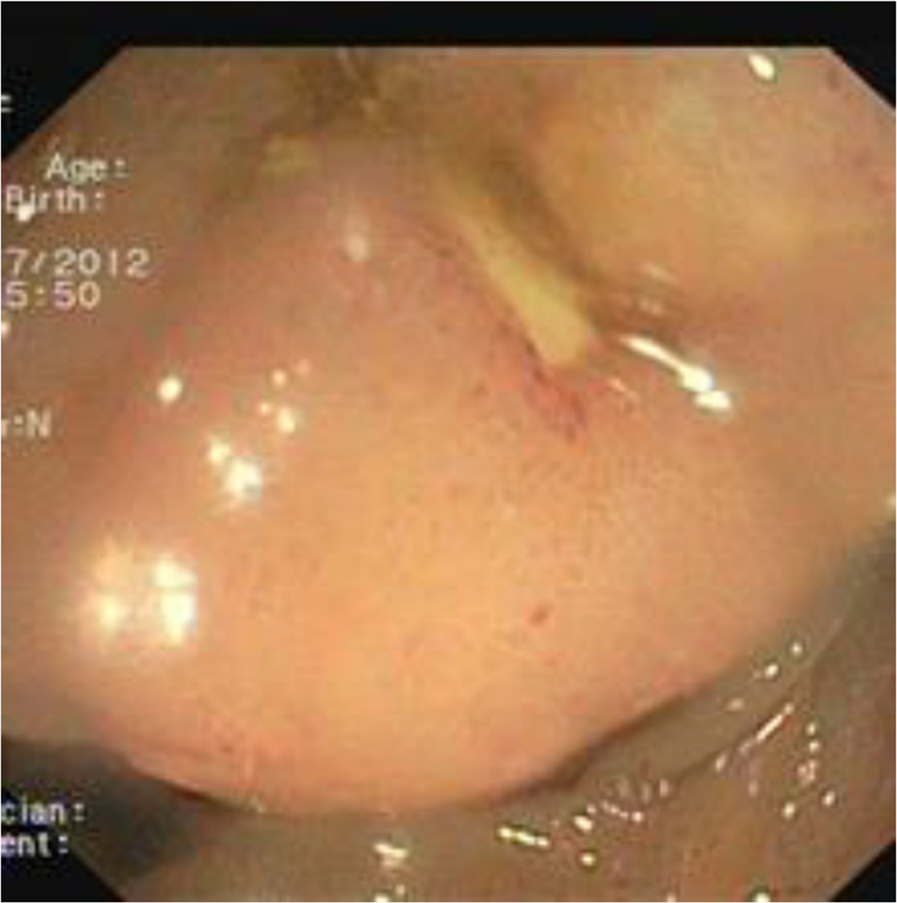
Colonscopy showed external compression of the cecum and mucosa appears edematous with hemorrhagic ulcerations.

**Figure 2:**
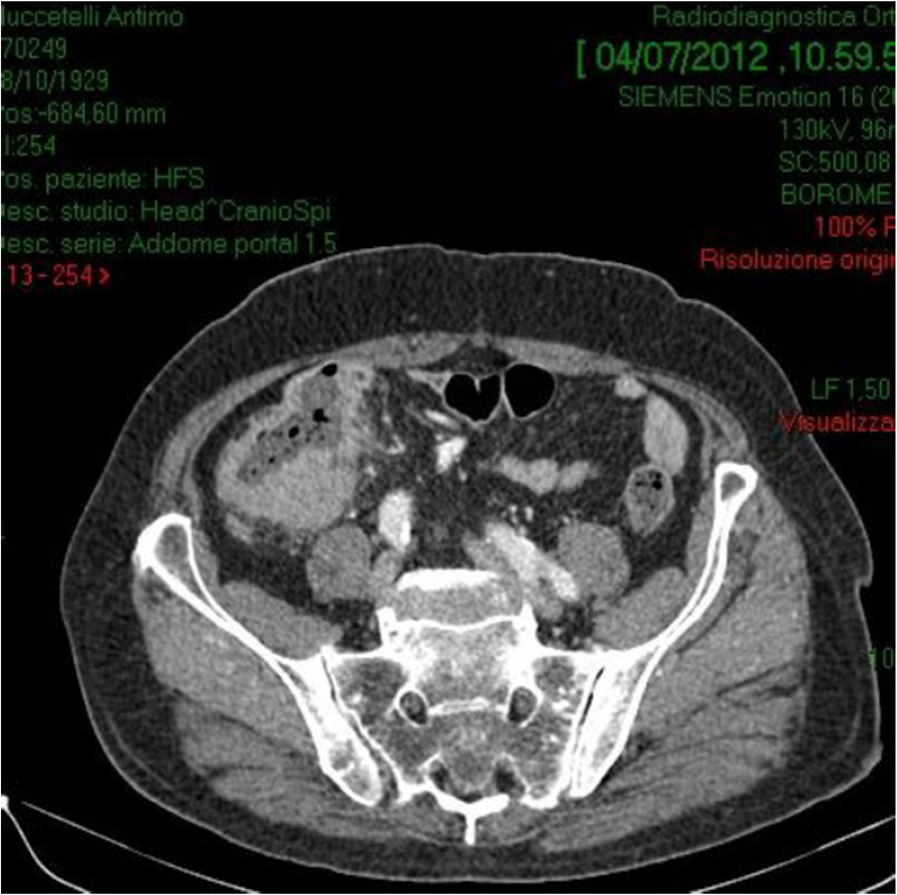
Abdominal computed tomography (CT) with intravenous contrast detected a ulcerated massat the proximal ascending right colon, with associated adenopathy of the ileo-colic pedicle.

**Figure 3:**
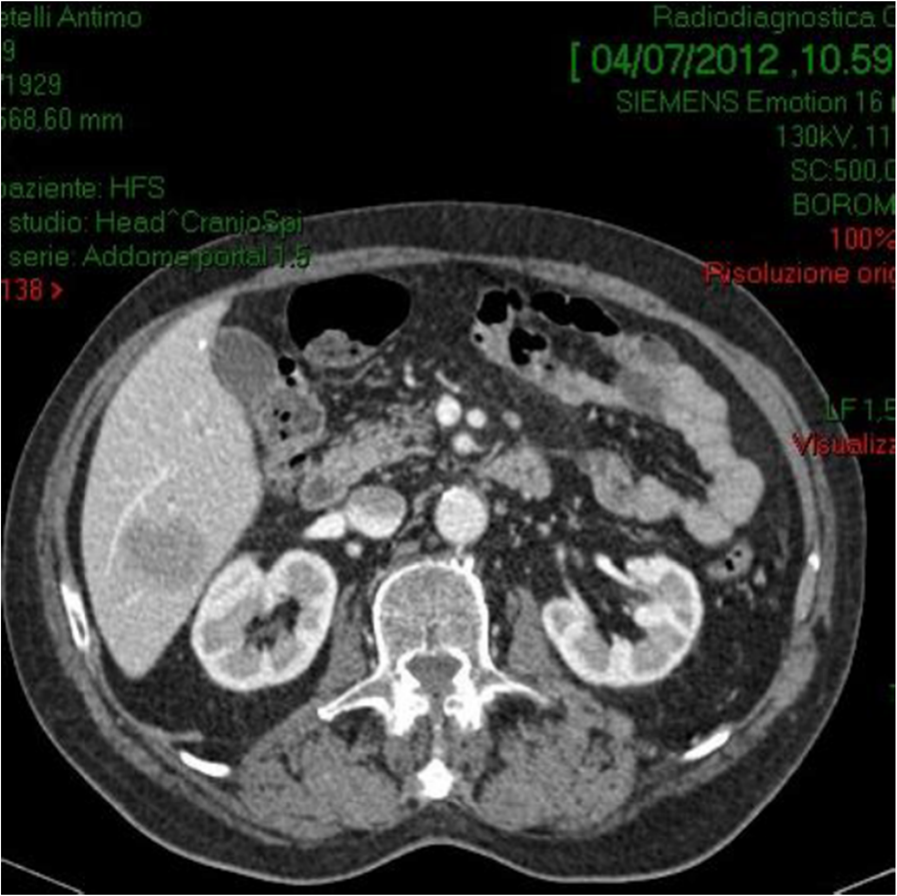
Hypovascular lesion with diameters of 3.5 cm suggestive for metastases from colorectal adenocarcinoma was detected in the liver segment 6 based on Couinaud’s classification.

A 70-year-old male presented with a 6-mo history of vague abdominal pain. The patient also complained of constipation, fatigue, weight loss and melena.The patient’s history included repaired right sided inguinal hernia, pulmonary fibrosis, hypertension and angina pectoris which was treated using coronary stents 5 months ago and dual antiplatelet therapy. He underwent a colonoscopy 6 years before, and the result was negative for colorectal diseases. The abdomen was soft, tender to palpation over the right and lower quadrant. Clinical examination on admission revealed the existence of a palpable mass in the right iliac fossa. Laboratory data, including tumor markers and leucocitary formula, were all within the normal limits, except for the hemoglobin level (hgb 8.8 g/dl). A colonoscopy revealed external compression of the cecum and mucosa appears edematous with two hemorrhagic ulcerations (Figure 1). Biopsies obtained during the colonoscopy were not diagnostic.Abdominal computed tomography (CT) with intravenous contrast showed a ulcerated mass at the proximal ascending right colon, with associated adenopathy of the ileo-colic pedicle and in the retroperitoneum (Figure 2). Hypovascular heterogeneous single lesion with diameters of 3.5 cm suggestive for metastases from colorectal adenocarcinoma was detected during the portal venous phase of liver enhancement in segment 6 based on Couinaud’s classification (Figure 3). The single liver lesion appears round and no radiological signs suggestive of vascular infiltration were present.

Computed tomography guided tru-cut biopsy of the liver nodule was not performed because of the rapid deterioration of the overall health status of patient. We didn't have repeat a new colonoscopy because of the patient's refusal.

During hospital stay, the patient presented a new episode of rectal bleeding and signs of hypovolemia such as tachycardia, hypotension, and low urine output. So blood transfusions were performed to replace blood lost.

Both the rapid health deterioration of patient and the high hemorragic risk associated with the risk of bowel obstruction prompted us to perform a right hemicolectomy associated with loco-regional lymphadenectomy. Under Pringle's maneuver a limited anatomic resection of segment 6 was performed.

**Figure 4:**
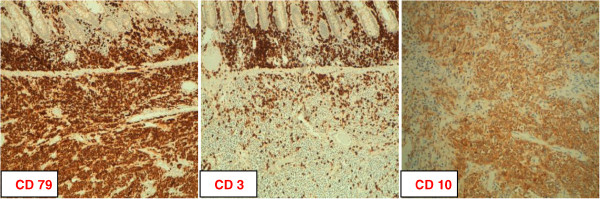
**Immunohistochemical profile of diffuse large B cell lymphoma of the cecum.** Diffuse large B-cell lymphoma cells were diffusely positive for CD 79, CD 3 and strongly positive for CD 10.

Gross examination of the resected specimen showed a distinct lesion in the cecum. Microscopic examination revealed diffuse large B cell lymphoma of the cecum and portions of colonic tissue infiltrated by the neoplasm.The cells were monotonous with irregular nuclear membranes and prominent nucleoli with easily found mitotic activity. Immunohistochemical staining was also performed and revealed the tumor to be CD10+, CD3+, CD79+ (Figure 4) and negative for BCL6 and MUM1.

The liver resected specimen revealed a metastasis of non-Hodgkin's lymphoma with central necrosis. Morphologic and immunohistochemical results confirmed the diagnosis of synchronous presentation of diffuse large B-cell lymphomas with mesenteric lymph nodes and a single liver metastasis. He was discharged home 12 days after his operation.

Four weeks after surgery, the patient underwent bone marrow biopsy for a further evaluation of the disease. Bone biopsy was negative for neoplastic involvement.

At 3-year follow-up, patient is alive and free of disease.

## Discussion

The colon is an uncommon site of involvement of Non-Hodgkin’s lymphoma (NHL). The most frequently involved colonic site at diagnosis is the ileocaecal region (approximately half of the cases), followed by the caecum, the sigmoid and the rectum. Clinical presentation of colonic lymphoma is quite vague and variable. The most common symptoms of colonic lymphoma are abdominal pain and weight loss with a palpable mass identified on physical examination. Primary colonic lymphomas usually occurs between the fourth and seventh decade of life.

The etiology of DLBCL is unknown, but some risk factors and predisposing conditions have been identified such as immunodeficienty and inflammatory bowel diseases [3]. Despite the severe luminal narrowing, lymphoma is less likely to cause obstruction because it does not elicit a desmoplastic response and submucosal lymphoid infiltration weakens the muscularis propria of the wall. However, its appearance may resemble that of other disease processes involving the cecum. The correctly diagnosis is often challenging and usually based on histological findings after operative colonic resection.

In general, the diagnosis of colonic lymphoma could be based on the CT appearance of extensive abdominal and/or pelvic lymphadenopathy. Even in the absence of lymphadenopathy, characteristic images such as location at the cecum, demarcation from the peri-colonic fat with no invasion of surrounding viscera and the presence of perforation in the absence of desmoplastic reaction should raise the suspicion of a lymphoma. Fisher et al. suggest that without the presence of enlarged lymph nodes it is difficult to distinguish this type of tumor from primary adenocarcinoma [4]. Radiologic examination of large bowel and colonoscopy with biopsy could not sufficient for definitive diagnosis of colonic lymphoma, but US and/or CT are invaluable for the staging of the disease. Malignant lymphoma of the colon has been reported in association with a variety of other entities, especially those of altered immune status and associations with chronic ulcerative colitis, Crohn’s disease, and celiac disease. Sciaudone et al. reported the presence of non-Hodgkin mantle cell lymphoma (MCL) with multiple lymphomatous polyposis (MLP) in a patient with a history of recurrent abdominal pain, diarrhea and rectal bleeding. They showed the importance of considering multiple lymphomatous polyposis in differential diagnosis of patients affected by atypical inflammatory bowel disease, because, although the clinical, radiological, endoscopic, and histological presentation of gastrointestinal MCL is very informative, it should be kept in mind that morphological features alone may not be sufficient to diagnose of colonic non-Hodgkin lymphoma [5].

Wang et al. showed that the morphology of primary colonic lymphoma (PCL) is variable, resulting in a wide range of endoscopic features, including mass lesions, narrowing of the lumen, ulceration, mucosal irregularities, and aphthous lesions [6]. Wyatt revealed that endoscopic findings of colonic lymphoma are classified by ulceration, infiltration, and the presence of a mass [7].

Furthermore for B-cell lymphomas, fungating mass is the most common endoscopic type (54.0%) [8].

The different presentations of colonic lymphoma could be better revealed by the use of double contrast barium enema technique. The radiologic changes may be similar to those found in Crohn's disease, amoebiasis or pseudomembranous colitis [9]; they can be divided into five groups: mucosal nodularity, endo-exoenteric mass, intraluminal mass, mural infiltration and mesenteric invasion [10].

The diagnosis of primary colonic lymphomas (PCL) was initially established in 1961 and included the following diagnostic five criteria [11]:

1. No palpable superficial lymphadenopathy;

2. No enlargement of the mediastinal lymph nodes on chest X-ray;

3. Normal white blood cell count;

4. Predominance of the bowel lesion, and adjacent lymph nodes affected at laparotomy;

5. Absence of any tumor in the liver or spleen at laparotomy.Our patient's disease met all these criteria preoperatively, except for the liver lesion detected by abdominal CT scan, but the radiologic characteristics of this lesion were similar to those of liver metastases from colorectal cancer. Abdominal computed tomography (CT) images revealed one hypovascular space-occupying lesions with diameters of 3.5 cm with internal heterogeneity due to a relative lack of effect of the contrast medium in the liver (Figure 3). This suggested the presence of metastasis releated to colon cancer. Furthermore it revealed enlarged lymph nodes around the stomach.

The most common histological subtype of colorectal lymphoma is diffuse large B-cell lymphoma (DLBCL), as our case reported [12]. Other histologies include follicular lymphoma, Burkitt lymphoma and Mantle cell lymphoma [13].

Colonic biopsies performed by our endoscopist were non-diagnostic and the diagnosis was made postoperatively after colonic resection. For definitive diagnosis is crucial to define morphology and immunophenotyping. Diffuse large B-cell lymphoma cells generally express pan B cell markers such as CD20, CD19, CD22, CD45 and CD79a; seventy percent of tumor cells express BCL-6 protein; CD10 is expressed in 30 to 60% of cases [14]. In our case, the tumour cells were strongly positive for CD10+ and CD3+. CD10 is considered to be a marker of follicular centre B-cell differentiation. Ohshima et al. reported that CD10 expression was closely associated with improved survival in patients with diffuse large B-cell lymphomas. So they concluded that that CD10 expression may be useful, in combination with clinical parameters, for determining the prognosis of DLBC [15].

In our case, the lymphoma presented as a colonic mass on colonoscopy, but it was not obvious on CT examination. We didn't perform double contrast barium enema because of the patient's refuseal. Some authors have reported rare cases of emphysematous colitis [16] as the first clinical manifestation, and also masquerading Crohn’s disease [17]. A multidisciplinary tumor board should manage all aspects of care of patients with colonic lymphoma.

Future advancements in molecular genetics, tumor analysis, and immunohistochemistry could led to most effective management strategies for patients with colonic lymphoma.

Accurate staging of bone marrow status is crucial to optimize the therapeutic strategy. Bone marrow biopsy (BMB) remains the gold standard to determine bone marrow involvement (BMI) but has poor sensitivity because the sample size may be small or the BMI focal [18].

Treatment of colorectal lymphoma usually involves chemotherapy, radiation, surgery or a combination of these. Quayle et al. reported that radiation therapy might not be the preferred option for the treatment of colonic lymphoma, because of a high risk of complications involving the small and large bowels [19]. Nowadays, due to the introduction of new active drugs as monoclonal antibodies like rituximab as part of chemotherapy treatment, the role of surgery is debatable. Some authors propose that surgery could be beneficial to prevent perforation or bleeding; on the other hand others suggest that early diagnosis and chemotherapy might avoid a surgical procedure. In our opinion, surgery may be beneficial in those patients at risk of complications such as hemorrhage, obstruction and perforation, but it should be associated with postoperative chemotherapy. Moreover, surgery alone with or without radiotherapy must be reserved for selected cases, as localized low-grade lymphomas, because the vast amount of lymphomas, moderate and high grade B cell lymphoma, generally extends beyond local fields. In these cases, chemotherapy, with or without surgical excision remains the basis of the treatment.

## Conclusion

This case is the first report of colonic lymphoma arising from the cecum with synchronous liver metastasis. It was impossible to achieve a correct preoperative diagnosis and the indication for surgery was supported by the high hemorrhagic risk associated with the risk of bowel obstruction and by rapid deterioration of the overall health status. In our case, both tomography and colonscopy showed findings mimicking colonic adenocarcinoma.

Colonic lymphoma is extremely rare and the variable imaging tests are non-specific; the diagnosis is rarely made before surgery and usually confirmed by histopathological investigation after surgery. This kind of tumour should be considered in the differential diagnoses of colon mass with with enlarged lymph nodes in the abdomen or pelvis and synchronous liver nodules when the biopsies obtained during the colonoscopy were not diagnostic.

## Consent

Written informed consent was obtained from the patient for publication of this Case report and any accompanying images. A copy of the written consent is available for review by the Editor of this journal.

## Competing interests

The authors declare that no conflict of interest to declare.

## Authors’ contributions

PI and ML performed the surgery and has given final approval of the version to be published; DR participated to surgery and wrote the manuscript; RP conducted a literature search and contributed to drafting the manuscript; AD, and FC participated in the acquisition and interpretation of radiological data; DA and CM carried out the histological and bio-molecular studies; LN and RC supervised the draft and cared the patien’s follow-up. All authors read and approved the final manuscript.

## Pre-publication history

The pre-publication history for this paper can be accessed here:

http://www.biomedcentral.com/1471-2482/14/75/prepub
